# Maternal exposure to ambient fine particulate matter and fetal growth in Shanghai, China

**DOI:** 10.1186/s12940-019-0485-3

**Published:** 2019-05-16

**Authors:** Zhijuan Cao, Lulu Meng, Yan Zhao, Chao Liu, Yingying Yang, Xiujuan Su, Qingyan Fu, Dongfang Wang, Jing Hua

**Affiliations:** 1 Department of Women and Children’s Health Care, Shanghai First Maternity and Infant Hospital, Tongji University School of Medicine, Shanghai, China; 2 College of Architecture and Urban Planning, Tongji University, Siping Rd. 1239, Shanghai, 200082 China; 3 Shanghai Environmental Monitoring Centre, Shanghai, China

**Keywords:** PM_2.5_, Ultrasound measures, Maternal exposure, Fetal growth restriction, China

## Abstract

**Background:**

Fetal growth restriction (FGR) is not only a major determinant of perinatal morbidity and mortality but also leads to adverse health effects in later life. Over the past decade, numerous studies have indicated that maternal exposure to ambient air pollution has been a risk factor for abnormal fetal growth in developed countries where PM_2.5_ levels are relatively low. However, studies in highly polluted regions, such as China, and studies that rely on assessments in utero are scarce.

**Methods:**

A total of 7965 women were selected from 11,441 women from the Shanghai Maternity and Infant Living Environment (SMILE) cohort who were pregnant between January 1, 2014, and April 30, 2015. From January 1, 2014, to April 30, 2015, weekly average PM_2.5_ values from 53 monitors were calculated and the inverse distance weighted (IDW) method was used to create a Shanghai pollution surface map according to the participants residential addresses. Individual exposure was the average PM_2.5_ value of every gestational week between the first gestational week and one week before the ultrasound measurement date (the range of measurements per participant was 1 to 10). Repeated fetal ultrasound measurements during gestational weeks 14~40 were selected. The estimated fetal weight (EFW) was calculated by biparietal diameter (BPD), abdominal circumference (AC), and femur length (FL) formulas. In total, 29,926 ultrasound measurements were analysed. Demographic variables, other pollutants (SO_2_, NO_2_, PM_10_ and O_3_) and relative humidity and temperature were controlled for potential confounding through generalized estimating equations (GEE).

**Results:**

The full model showed that with each 10 μg/m^3^ increase in PM_2.5_ exposure, the means (mm) of AC, BPD, FL decreased by 5.48 (− 9.06, − 1.91), 5.57 (− 6.66, − 4.47), and 5.47 (− 6.39, − 4.55), respectively; the mean EFW decreased by 14.49 (− 16.05, − 13.49) grams by Hadlock’s third formula and 13.56 (− 14.71, − 12.50) grams by Shepard’s formula with each 10 μg/m^3^ increase in PM_2.5_ exposure.

**Conclusions:**

A negative correlation existed between maternal PM_2.5_ exposure during pregnancy and fetal growth indicators, which may increase the risk of fetal growth restriction.

**Electronic supplementary material:**

The online version of this article (10.1186/s12940-019-0485-3) contains supplementary material, which is available to authorized users.

## Background

Fetal growth restriction (FGR) is a pathologic condition in which the fetus fails to reach its biologically-based growth potential [1]. The condition is believed to be a major determinant of perinatal and childhood morbidity and mortality [2, 3]. FGR may also cause a predisposition to a range of diseases later in life, particularly cardiovascular and metabolic diseases [3, 4]. Normal fetal growth depends on a complex combination of genetic, social, and environmental factors [5]. Over the past decade, numerous studies have indicated that maternal exposure to ambient air pollution has been a risk factor for abnormal fetal growth [6–9].

Most studies rely on assessments at birth, yet these do not adequately capture in utero growth patterns over the course of the pregnancy as growth is dynamic, not static, and more than one measurement is necessary to make a prospective determination of (impaired) growth [10]. Some growth impairment and imbalance that may occur during the early period may be compensated for in the remaining pregnancy period [11]. Assessing fetal growth in utero using repeated ultrasound measures could provide a more accurate classification of restricted growth by reducing the time between exposure and outcome assessment [11].

To date, a small number of studies have investigated the association between exposure to various air pollutants (i.e., NO_2_, SO_2_, O_3_, CO and PM_10_) during pregnancy and fetal growth measured by ultrasound [12–17]. Most of them were conducted in Europe or the USA, where PM_2.5_ levels are relatively low. Studies in highly polluted regions, such as China, can further elucidate the magnitude of PM_2.5_-associated health effects at high exposure levels. However, because of the lack of ground measurement systems for PM_2.5_ and the limited spatial representativeness of measurements from central ground monitors in China, no study has analysed the association between ultrasound measures of fetal growth and maternal exposure to PM_2.5_ during the fetal period.

The recent establishment of air quality monitoring networks makes it possible to investigate prenatal exposure to PM_2.5_ and the risk of FGR in China. We analysed data from the Shanghai Maternity and Infant Living Environment (SMILE) cohort to test the hypothesis that whether exposure to high levels of PM_2.5_ during pregnancy could increase the risk of FGR.

## Methods

### Study population

This study was embedded in the SMILE cohort, which has been established in the Shanghai First Maternity and Infant Hospital since January 2014. All voluntary pregnant women who receive routine antenatal care during pregnancy and deliver at the Shanghai First Maternity and Infant Hospital are enrolled in the SMILE cohort. Participants were interviewed at the obstetric clinic. Personal information regarding specific residential address, maternal age, maternal height, pre-pregnancy weight, reproductive and medical histories, maternal parity and gravidity were collected. The Human Ethics Committee of Shanghai First Maternity and Infant Hospital approved the study, and all participating mothers provided written informed consent prior to participation.

In the current study, participants in the SMILE cohort who were pregnant (the first day of the last menstrual period was considered the date of pregnancy [18]) between January 1, 2014 and April 30, 2015 were selected. To control biases, subjects meeting one or more of the following criteria were excluded: a. living in Shanghai for less than three years; b. suffering from serious diseases (diabetes, hypertension, hyperthyroidism, tumour, epilepsy, etc.) before the current pregnancy; c. used assisted reproductive technology for this pregnancy; d. having twins or multiples; e. experienced termination of pregnancy, stillbirth.

### PM_2.5_ exposure assessment

**Fig. 1:**
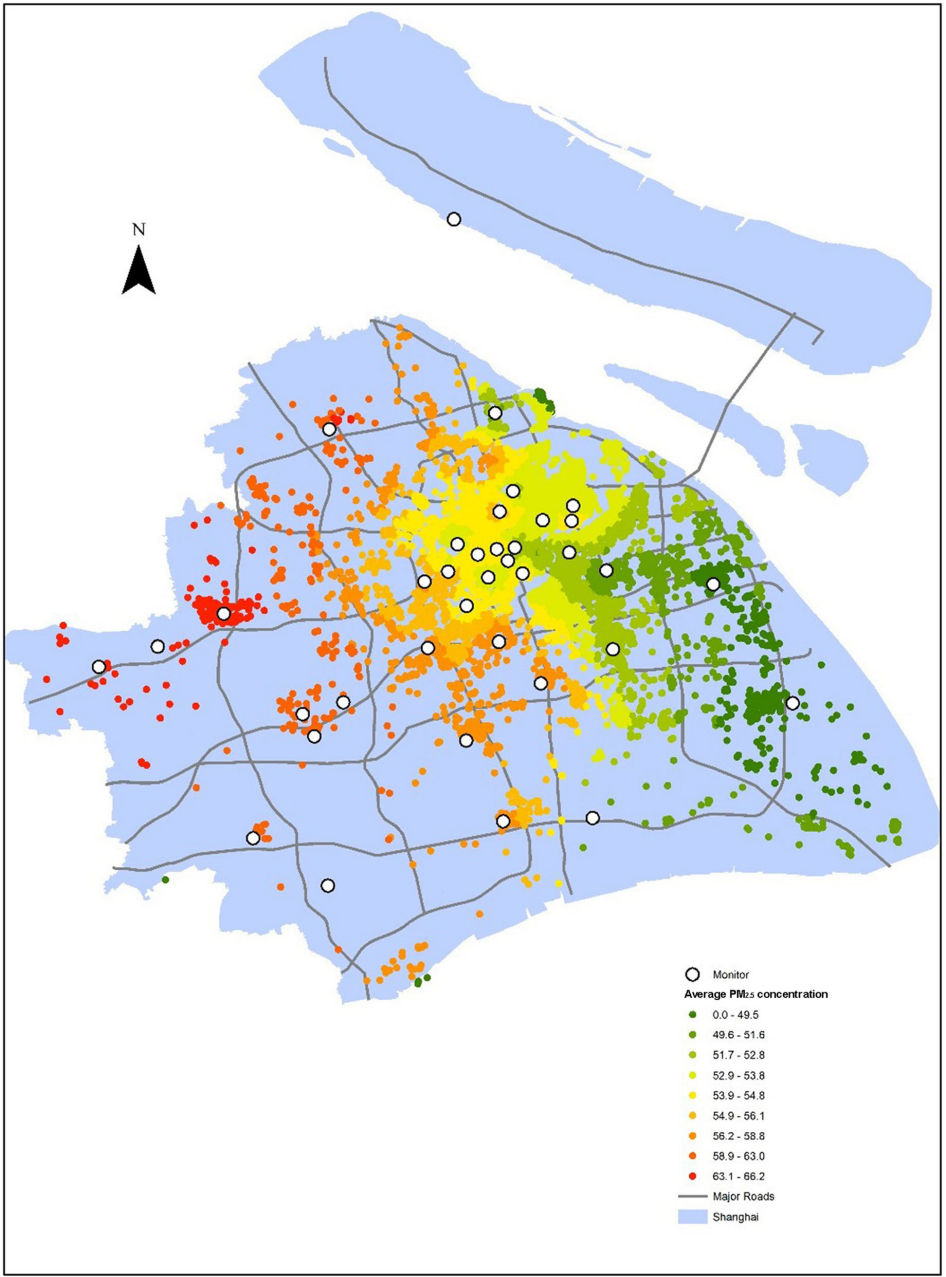
Monitor distributions and PM_2.5_ pollution surface map in Shanghai

Shanghai is a coastal city located in the middle eastern part of China (Fig. 1). The study area included sixteen districts in the main city of Shanghai, excluding Chongming District. The Chongming District was excluded because it is an isolated island at the mouth of the Yangtze River that does not share similarities with mainland patterns. Overall, the study area covered 5515 km^2^ of land and 23.1 million people in the mainland of Shanghai. Shanghai began to monitor PM_2.5_ concentrations in 2012, and new monitors have been gradually added over the past three years. By the end of 2014, there were ten national-standard monitoring stations reporting monitor data hourly in the city and forty-four regional-standard monitoring stations. As shown in Fig. 1, these stations cover every municipal district in Shanghai; however, they are distributed more densely in urban areas.

Interpolation is a procedure to predict the value of attributes at non-sampled sites from measurements made at point locations within the same area [19]. A proper interpolation method for PM_2.5_ concentration should be selected before calculating fine resolution excess health burdens. Three interpolating methods are commonly used to transform PM_2.5_ data into concentration maps: closest monitor (CM), inverse distance weighted (IDW), and Voronoi neighbourhood averaging (VNA) interpolation methods [20]. IDW has the lowest root mean square error (RMSE) based on leave-one-out cross-validation (LOOCV) evaluation of the three methods [21]. Therefore, IDW was chosen to estimate maternal exposure to PM_2.5_ at sub-district levels. IDW is a deterministic, nonlinear interpolation method using a weighted average of the attribute values from nearby points to estimate the value at non-sampled locations to create a value surface. An inverse square of distance is mostly employed in surface interpolation and was also applied in this paper.

In this study, we used monitored air pollution data from the Shanghai Environment Monitoring Centre. The daily PM_2.5_ pollution data were collected from January 1, 2014 to April 30, 2015 from 53 monitors. Every seven daily records were averaged to produce weekly mean values. Then, weekly PM_2.5_ values were interpolated to cover all the whole of Shanghai by the IDW method to create the Shanghai pollution surface map (Fig. 1) according to the participating residential address (at the beginning of pregnancy). Individual exposure was the average PM_2.5_ value of every gestational week between the first gestational week and one week before the ultrasound measurement date. At the same time, the average values of other pollutants (SO_2_, NO_2_, PM_10_ and O_3_) and meteorological factors (relative humidity and temperature) throughout pregnancy from 53 monitoring sites were controlled for confounding.

### Fetal growth assessment

Ultrasound is widely used worldwide to detect abnormal fetal growth. The repeated ultrasound measurements of abdomen circumference (AC), femur length (FL) and biparietal diameter (BPD) during pregnancy were collected from the medical records. All records were recorded in millimetres. The estimated fetal weight (EFW) was calculated by two of the most widely accepted formulas, Hadlock’s third formula and Shepard’s formula [22]. Because the fetal size cannot be accurately assessed during the first trimester, fetal ultrasound measurements during the second trimester and third trimester (14–40 gestational weeks) were selected only [18]. A total of 29,926 ultrasound records were analysed.

### Statistical analyses

A generalized estimating equation (GEE) was used to analyse the repeated measurements. The continuous measurements of AC, FL, BPD and EFW were dependent variables in the GEE linear model. To better control the confounding factors, three kinds of confounders were controlled stepwise. They were demographic variables (gestational age, infant gender, gestational hypertension, gestational diabetes, mother age, parity, gravidity, staff medical insurance and season of conception), other pollutants (SO_2_, NO_2_, PM_10_ and O_3_) and meteorological factors (relative humidity and temperature). In the crude model, only gestational age was controlled; in the adjusted-1 model, all demographic variables were controlled; in the adjusted-2 model, demographic variables and other pollutants were both controlled; in the adjusted-3 model (the full model), demographic variables, other pollutants and meteorological factors were controlled. Sensitivity analyses were conducted for preterm deliveries (gestational age less than 37 weeks), gender, gestational diabetes and gestational hypertension respectively. All analyses were performed using Stata version 15.0, and all tests were two-sided.

## Results

A total of 7965 women were selected from 11,441 pregnant women from the SMILE cohort who were pregnant between January 1, 2014, and April 30, 2015. Maternal age, registered residence, parity, gravidity, staff medical insurance, gestational hypertension, gestational diabetes mellitus, body mass index pre-pregnancy, height, weight pre-pregnancy, newborn gender, season of conception, preterm birth and low birth weight are described in Table 1.

**Table 1:** Distributions of selected characteristics of the study population

Characteristics	N (%)
Maternal age (years)	
22–28	2783 (34.9)
29–35	4516 (56.7)
> 35	666 (8.36)
Registered residence (missing = 1)	
Permanent	4755 (59.7)
Migrant	3209 (40.3)
Parity (missing = 1)	
1	6405 (80.4)
2	1559 (19.6)
Gravidity (missing = 6)	
1	5076 (63. 8)
≥ 2	2883 (36.2)
SMI^a^ (missing = 28)	
No	2448 (30.8)
Yes	5489 (69.2)
GH^b^	
No	7567 (95.0)
Yes	398 (5.0)
GDM^c^	
No	6771 (85.0)
Yes	1194 (15.0)
BMI (pre-pregnancy)	
Under weight	573 (7.2)
Normal weight	6129 (77.0)
Overweight	1062 (13.3)
Obesity	201 (2.5)
Height (mean ± sd)/cm	161.67 ± 4.44
Weight (pre-pregnancy) (mean ± sd)/kg	57.11 ± 11.99
Newborn gender	
Male	4045 (50. 8)
Female	3920 (49.2)
Season of conception	
Spring	2216 (27.8)
Summer	1839 (23.1)
Autumn	1756 (22.1)
Winter	2154 (27.0)
PTB^d^	
No	7586 (95.2)
Yes	379 (4.8)
LBW^e^	
No	7732 (97.1)
Yes	233 (2.9)

^a^Staff Medical Insurance; ^b^Gestational Hypertension; ^c^Gestational Diabetes Mellitus; ^d^Preterm Birth; ^e^Low Birth Weight

The concentration distribution of atmospheric pollutants throughout pregnancy is described in Table 2. The average concentration of individual PM_2.5_ exposure was 51.20 μg/m^3,^ with a minimum of 29.05 μg/m^3^ and a maximum of 82.10 μg/m^3^. The correlations between air pollutants, temperature and relative humidity are shown in the Additional file 1.

**Table 2:** Description of pollutants’ concentration (μg/m^3^) throughout pregnancy

Pollutants	Mean	Percentiles			Range
		25	50	75	
PM_2.5_	48.0	44.5	48.6	51.2	34.3~67.9
PM_10_	60.5	55.8	62.6	64.4	36.7~80.6
NO_2_	42.3	36.8	44.2	50.1	8.7~55.9
O_3_	67.5	63.1	66.8	73.3	0.7~87.1
SO_2_	16.5	14.2	17.4	18. 6	1.1~22.2
RH^a^	17.9	15.4	18.7	20.2	9.0~23.8
T^b^	70.1	69.7	70.2	70.7	65.4~73.5
Ie (PM_2.5_) ^c^	51.2	46.6	50.5	55.0	29.1~82.1

^a^Relative humidity;

^b^Temperature;

^c^Individual exposure, it was the average PM_2.5_ values of every gestational week between the first gestational week to the one week before the ultrasound measurement date

A total of 29,926 ultrasound examinations were performed on 7965 pregnant women. The number of ultrasound measurements per pregnant woman ranged from 1 to 10. The average gestational age for the first and second ultrasound measurements was in the second trimester, and the average gestational age of the remaining ultrasound examinations was in the third trimester. Table 3 shows the gestational age distribution of each time of ultrasound measurement in detail.

**Table 3:** Summary of gestational age at each time of ultrasound measurement

Ultrasound measurements	Summary of gestational age			
	Mean	Std. Dev	Freq	Rang
1st	19.96	5.15	7965	13.5~40.0
2nd	25.48	4.28	7269	13.6~40.6
3rd	30.93	4.25	6280	14.1~40.4
4th	34.91	3.55	4701	15.2~40.6
5th	36.97	3.04	2540	14.1~40.7
6th	37.69	2.91	850	14.0~40.9
7th	37.59	3.28	235	14.3~40.6
8th	37.35	3.32	55	24.0~40.6
9th	37.25	3.37	21	28.6~40.6
10th	37.44	2.17	10	32.4~40.4
Total	28.09	7.54	29,926	13.5~40.9

We observed negative associations of prenatal PM_2.5_ exposure with the continuous measures of fetal growth (Table 4). In the full model, with each 10 μg/m^3^ increase in PM_2.5_ exposure, the means (mm) of AC, BPD, FL decreases by 5.48 (−9.06, −1.91), 5.57 (−6.66, −4.47) and 5.47 (− 6.39, − 4.55), respectively. The effect coefficient for all the pollutants and the meteorological factors were shown in the Additional file 2. The mean EFW decreased by 14.49 (− 16.05, − 13.49) grams by Hadlock’s third formula and 13.56(− 14.71, − 12.50) grams by Shepard’s formula with each 10 μg/m^3^ increase in PM_2.5_ exposure in the full model.

**Table 4:** The association between PM_2.5_ exposure during pregnancy and ultrasound measures for fetal growth

Fetal growth parameters	β^a^ (95% CI)			
	Crude ^b^	Adjusted-1 ^c^	Adjusted-2 ^d^	Adjusted-3(the full model) ^e^
AC	−0.62(−3.35, 2.11)	−5.71(− 9.17, − 2.25)	−5.57(− 9.06, − 2.08)	−5.48(− 9.06, − 1.91)
BPD	−1.63(− 2.48, − 0.78)	−5.82(− 6.88, − 4.75)	−5.77(− 6.84, − 4.69)	−5.57(− 6.66, − 4.47)
FL	−2.21(− 2.91, − 1.50)	− 5.82(− 6.71, − 4.92)	−5.73(− 6.63, − 4.83)	−5.47(− 6.39, − 4.55)
EFW-H ^f^	−18.11(− 19.33, − 16.96)	−14.45(− 16.96, − 15.72)	−14.53(− 15.81, − 13.34)	−14.49(− 16.05, − 13.49)
EFW-S ^g^	−16.63(− 17.68, − 15.63)	−13.40(− 14.50, − 12.38)	−13.44(− 14.56, − 12.41)	−13.56(− 14.71, − 12.50)

^a^estimates per 10 μg/m3 increase in PM_2.5_ exposure.

^b^Adjusted for gestational age.

^c^Adjusted for a and (infants’ gender, pregnancy hypertension, gestational diabetes, mother age, parity, gravidity, staff medical insurance, season of conception).

^d^Adjusted for a, b and (SO_2_, NO_2_, PM_10_ and O_3_).

^e^Adjusted for a, b, c and (relative humidity and temperature).

^f^Estimated fetal weight calculated by Hadlock’s third formula: Log10(EFW-H) =1.304 + (0.05281*AC) + (0.1938*FL) -(0.004*AC*FL).

^g^Estimated fetal weight calculated by Shepard formula: Log10(EFW-S) =1.2508 + (0.166*BPD) + (0.046*AC) -(0.002646*AC*BPD).

**Fig. 2:**
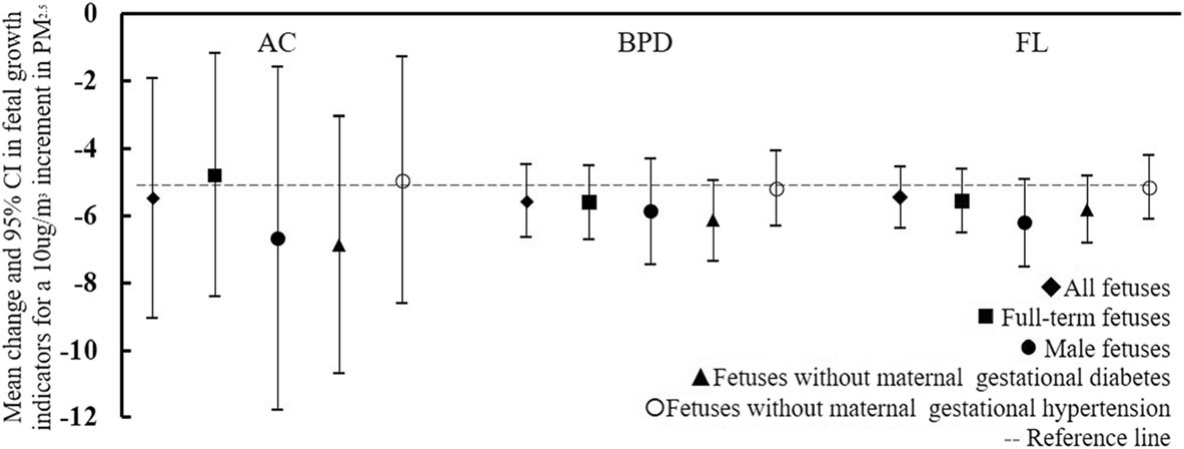
Sensitivity analyses for preterm deliveries, gender, gestational diabetes and gestational hypertension. Note: Sensitivity analyses were conducted in the full model

Results of sensitivity analyses for preterm deliveries (gestational age less than 37 weeks), gender, gestational diabetes and gestational hypertension presented that all 95% confidence interval of three fetal growth indicators were partially overlapping with the results in all fetuses (Fig. 2).

## Discussion

FGR is a complex and multifactorial disorder affecting fetal development that often results in multiple perinatal complications [23] and currently represents a major risk factor for poor long-term neurological outcomes [24]. However, at present, there is no effective treatment to reverse the course of FGR except delivery [23]. Therefore, the prevention of FGR is even more important. Due to the lack of a standardized definition of FGR, we cannot draw a direct correlation between prenatal PM_2.5_ exposure and FGR, but AC, FL and BPD are important indicators for EFW, which can indirectly reflect the association between prenatal PM_2.5_ exposure and FGR. Consistent with previous similar studies [6, 8, 11, 12, 25, 26], our study showed that atmospheric PM_2.5_ exposure during pregnancy negatively affected all measures of fetal growth. However, the effect coefficients were much higher than those in previous studies, which may be due to the higher atmospheric PM_2.5_ concentration in China.

The results showed that PM_2.5_ had similar impacts on AC, BPD and FL. AC is closely related to fetal fat development, and BPD and FL are more closely related to bone development. Previous studies have revealed that PM_2.5_ exposure may affect adipose tissue [27, 28] in mice and bone development [29] in adults; however, no studies have revealed the mechanism in human fetuses, and further studies are needed to explore the mechanism.

As sensitivity analyses, we excluded preterm deliveries (gestational age less than 37 weeks), maternal gestational diabetes fetuses and maternal gestational hypertension fetuses, respectively. The results were similar to those in the whole population. Inconsistent with other studies [30], we did not find a difference in the effects on fetal growth caused by atmospheric PM_2.5_ exposure during pregnancy between male fetuses and all fetuses. Additionally, fetal growth was analysed in consecutive weeks of gestation rather than in each trimester by repeated ultrasonic measurements. The present study is the first to reveal the potential adverse effects of maternal exposure to ambient PM_2.5_ on fetal growth before birth in China and to further elucidate the magnitude of PM_2.5_-associated health effects at high exposure levels.

There were some limitations to this study. First, the maternal active and passive smoking status were not investigated. However, according to a previous study, the proportion of pregnant women who actively smoke is very low (nearly 2%) [31], and the passive rate during pregnancy was 7.8% [32] in Shanghai, China. Second, we were unable to determine more detailed maternal activity patterns beyond simple residential location, and thus, exposure estimates will suffer some misclassification. However, much of the mismeasurement is likely to be random in terms of pollution exposure. Third, socioeconomic status (SES) was not collected. Individuals with a deferent SES background may take different protection behaviours for air pollution (i.e., wearing masks and using air purifiers). In this study, registered residence and staff medical insurance were used to reflect the SES level indirectly.

## Conclusion

A negative correlation existed between maternal PM_2.5_ exposure during pregnancy and fetal growth indicators, which may increase the risk of fetal growth restriction.

## Additional files

Additional file 1: **Table S1.** The correlations between air pollutants, temperature and relative humidity. (DOCX 15 kb)

Additional file 2: **Table S2.** Effect coefficient for all the pollutants and the meteorological factors in the full model. (DOCX 17 kb)

## Abbreviations

AC: Abdominal circumference
BPD: Biparietal diameter
CM: Closest monitor
EFW: Estimated fetal weight
FGR: Fetal growth restriction
FL: Femurlength
GEE: Generalized estimating equations
IDW: Inverse distance weighted
LOOCV: Leave-one-out cross-validation
PM_2.5_: Atmospheric fine particulate matter
RMSE: Root mean square error
SES: Socioeconomic status
SMILE: Shanghai Maternity and Infant Living Environment
VNA: Voronoi neighbourhood averaging

## References

[1] Gordijn SJ, Beune IM, Ganzevoort W (2018). Building consensus and standards in fetal growth restriction studies. Best Pract Res Clin Obstet Gynaecol.

[2] Gordijn SJ, Beune IM, Thilaganathan B (2016). Consensus definition of fetal growth restriction: a Delphi procedure. Ultrasound Obst Gyn.

[3] Smith G (2018). Universal screening for fetal growth restriction. Best Pract Res Clin Obstet Gynaecol..

[4] Liu S, Krewski D, Shi Y, Chen Y, Burnett RT (2007). Association between maternal exposure to ambient air pollutants during pregnancy and fetal growth restriction. J Expo Sci Environ Epidemiol.

[5] Zhu X, Liu Y, Chen Y, Yao C, Che Z, Cao J (2015). Maternal exposure to fine particulate matter (PM2.5) and pregnancy outcomes: a meta-analysis. Environ Sci Pollut R.

[6] Percy Z, DeFranco E, Xu F, et al. Trimester specific PM2. 5 exposure and fetal growth in Ohio, 2007–2010. J Environ Res. 2019;171:111–18. 10.1016/j.envres.2019.01.031. 10.1016/j.envres.2019.01.031 PMC6382528 30660917

[7] Symanski E, Davila M, McHugh MK, Waller DK, Zhang X, Lai D (2014). Maternal exposure to fine particulate pollution during narrow gestational periods and newborn health in Harris County, Texas. Matern Child Health J.

[8] Ritz B, Qiu J, Lee P (2014). Prenatal air pollution exposure and ultrasound measures of fetal growth in Los Angeles, California. Environ Res.

[9] Smarr MM, Vadillo-Ortega F, Castillo-Castrejon M, O Neill MS (2013). The use of ultrasound measurements in environmental epidemiological studies of air pollution and fetal growth. Curr Opin Pediatr.

[10] Golan R, Kloog I, Almog R (2018). Environmental exposures and fetal growth: the Haifa pregnancy cohort study. BMC Public Health.

[11] Zhao N, Qiu J, Ma S (2018). Effects of prenatal exposure to ambient air pollutant PM10 on ultrasound-measured fetal growth. Int J Epidemiol.

[12] van den Hooven EH, Pierik FH, de Kluizenaar Y (2012). Air pollution exposure during pregnancy, ultrasound measures of fetal growth, and adverse birth outcomes: a prospective cohort study. Environ Health Perspect.

[13] Malmqvist E, Liew Z, Kallen K (2017). Fetal growth and air pollution - a study on ultrasound and birth measures. Environ Res.

[14] Slama R, Thiebaugeorges O, Goua V (2009). Maternal personal exposure to airborne benzene and intrauterine growth. Environ Health Perspect.

[15] Iniguez C, Esplugues A, Sunyer J (2016). Prenatal exposure to NO2 and ultrasound measures of fetal growth in the Spanish INMA cohort. Environ Health Perspect.

[16] Aguilera I, Garcia-Esteban R, Iniguez C (2010). Prenatal exposure to traffic-related air pollution and ultrasound measures of fetal growth in the INMA Sabadell cohort. Environ Health Perspect.

[17] Hansen CA, Barnett AG, Pritchard G (2008). The effect of ambient air pollution during early pregnancy on fetal ultrasonic measurements during mid-pregnancy. Environ Health Perspect.

[18] Papageorghiou AT, Ohuma EO, Gravett MG (2016). International standards for symphysis-fundal height based on serial measurements from the fetal growth longitudinal study of the INTERGROWTH-21st project: prospective cohort study in eight countries. BMJ..

[19] Sajjadi SA, Zolfaghari G, Adab H, Allahabadi A, Delsouz M (2017). Measurement and modeling of particulate matter concentrations: applying spatial analysis and regression techniques to assess air quality. MethodsX..

[20] Reyes JM, Xu Y, Vizuete W, Serre ML (2017). Regionalized PM2. 5 community multiscale air quality model performance evaluation across a continuous spatiotemporal domain. Atmos Environ.

[21] Li L, Losser T, Yorke C, Piltner R (2014). Fast inverse distance weighting-based spatiotemporal interpolation: a web-based application of interpolating daily fine particulate matter PM2:5 in the contiguous U.S. using parallel programming and k-d tree. Int J Environ Res Public Health.

[22] Hadlock FP, Harrist RB, Sharman RS, Deter RL, Park SK (1985). Estimation of fetal weight with the use of head, body, and femur measurements--a prospective study. Am J Obstet Gynecol.

[23] Dall'Asta A, Brunelli V, Prefumo F, Frusca T, Lees CC (2017). Early onset fetal growth restriction. Matern Health Neonatol Perinatol.

[24] Colella M, Frérot A, Novais ARB, Baud O (2018). Neonatal and long-term consequences of fetal growth restriction. Curr Pediatr Rev.

[25] Lamichhane DK, Ryu J, Leem J (2018). Air pollution exposure during pregnancy and ultrasound and birth measures of fetal growth: a prospective cohort study in Korea. Sci Total Environ.

[26] Clemens T, Turner S, Dibben C (2017). Maternal exposure to ambient air pollution and fetal growth in north-East Scotland: a population-based study using routine ultrasound scans. Environ Int.

[27] Xu Z, Xu X, Zhong M (2011). Ambient particulate air pollution induces oxidative stress and alterations of mitochondria and gene expression in brown and white adipose tissues. Part Fibre Toxicol.

[28] Mendez R, Zheng Z, Fan Z, Rajagopalan S, Sun Q, Zhang K (2013). Exposure to fine airborne particulate matter induces macrophage infiltration, unfolded protein response, and lipid deposition in white adipose tissue. Am J Transl Res.

[29] Schraufnagel DE, Balmes J, Cowl CT (2019). Air pollution and non-communicable diseases: a review by the forum of international respiratory societies’ environmental committee. Part 2: air pollution and organ systems. Chest.

[30] Winckelmans E, Vrijens K, Tsamou M (2017). Newborn sex-specific transcriptome signatures and gestational exposure to fine particles: findings from the ENVIR ON AGE birth cohort. Environ Health.

[31] Li T, Yuan W, Li J, Song M, Zhou Q, Su X (2016). Prevalence of maternal passive smoking and its impact on pregnancy following implementation of an anti-smoking legislation in Shanghai, China: a cross-sectional study. Int J Clin Exp Med.

[32] Shi L, Dong Y, Pei C (2017). Passive smoking status and its influencing factors among pregnant women in Shanghai. J Shanghai Jiaotong University (Med Sci).

