# Supplementary material for: Maternal exposure to ambient fine particulate matter and fetal growth in Shanghai, China
Source: Environ Health. 2019 May 16;18:49. doi: 10.1186/s12940-019-0485-3 (PMC6524254; doi:10.1186/s12940-019-0485-3)
Supplement: Supplementary file 1 — Table S1. The correlations between air pollutants, temperature and relative humidity. (DOCX 15 kb) [file 12940_2019_485_MOESM1_ESM.docx]

Supplementary Table 1: The correlations between air pollutants, temperature and relative humidity

| Correlation coefficient | PM_2.5_ | SO_2_ | PM_10_ | NO_2_ | RH^a^ | O_3_ | T^b^ |
| --- | --- | --- | --- | --- | --- | --- | --- |
| PM_2.5_ | 1.00 |  |  |  |  |  |  |
| SO_2_ | 0.77^***^ | 1.00 |  |  |  |  |  |
| PM_10_ | 0.71^***^ | 0.73^***^ | 1.00 |  |  |  |  |
| NO_2_ | 0.34^***^ | 0.36^***^ | 0.35^***^ | 1.00 |  |  |  |
| RH^a^ | -0.85^***^ | -0.80^***^ | -0.68^***^ | -0.33^***^ | 1.00 |  |  |
| O_3_ | -0.57^***^ | -0.15^***^ | -0.52^***^ | -0.14^***^ | 0.39^***^ | 1.00 |  |
| T^b^ | -0.51^***^ | -0.50^***^ | -0.61^***^ | -0.24^***^ | 0.59^***^ | 0.19^***^ | 1.00 |

a. Relative humidity; b. Temperature; *** *p*<0.001.
