# Supplementary material for: Maternal exposure to ambient fine particulate matter and fetal growth in Shanghai, China
Source: Environ Health. 2019 May 16;18:49. doi: 10.1186/s12940-019-0485-3 (PMC6524254; doi:10.1186/s12940-019-0485-3)
Supplement: Supplementary file 2 — Table S2. Effect coefficient for all the pollutants and the meteorological factors in the full model. (DOCX 17 kb) [file 12940_2019_485_MOESM2_ESM.docx]

Supplementary Table 2: Effect coefficient for all the pollutants and the meteorological factors in the full model

| Dependent variable | Independent variables | Coef. | St. Err | t-value | p-value | Sig. |
| --- | --- | --- | --- | --- | --- | --- |
| AC | Ie (PM_2.5_) ^a^ | -5.483 | 1.824 | -3.01 | 0.003 | ^***^ |
|  | SO_2_ | 0.088 | 0.088 | 1.00 | 0.317 |  |
|  | NO_2_ | 0.016 | 0.007 | 2.28 | 0.022 | ^**^ |
|  | PM_10_ | -0.067 | 0.038 | -1.76 | 0.078 | ^*^ |
|  | O_3_ | -0.041 | 0.016 | -2.56 | 0.010 | ^**^ |
|  | T^b^ | -1.202 | 0.160 | -7.53 | 0.000 | ^***^ |
|  | RH^c^ | 0.451 | 0.144 | 3.13 | 0.002 | ^***^ |
| FL | Ie (PM_2.5_) | -5.471 | 0.470 | -11.63 | 0.000 | ^***^ |
|  | SO_2_ | 0.064 | 0.023 | 2.83 | 0.005 | ^***^ |
|  | NO_2_ | 0.001 | 0.002 | 0.72 | 0.472 |  |
|  | PM_10_ | -0.034 | 0.010 | -3.40 | 0.001 | ^***^ |
|  | O_3_ | -0.030 | 0.004 | -7.28 | 0.000 | ^***^ |
|  | T | -0.557 | 0.041 | -13.52 | 0.000 | ^***^ |
|  | RH | 0.228 | 0.036 | 6.42 | 0.000 | ^***^ |
| BPD | Ie (PM_2.5_) | -5.566 | 0.560 | -9.95 | 0.000 | ^***^ |
|  | SO_2_ | 0.090 | 0.027 | 3.36 | 0.001 | ^***^ |
|  | NO_2_ | 0.003 | 0.002 | 1.21 | 0.228 |  |
|  | PM_10_ | -0.041 | 0.012 | -3.50 | 0.000 | ^***^ |
|  | O_3_ | -0.028 | 0.005 | -5.70 | 0.000 | ^***^ |
|  | T | -0.693 | 0.049 | -14.14 | 0.000 | ^***^ |
|  | RH | 0.248 | 0.042 | 5.86 | 0.000 | ^***^ |

*** *p*<0.01, ** *p*<0.05, * *p*<0.1

a. Individual PM_2.5_ exposure

b. Temperature;

c. Relative humidity.
